# Induction of Thyroid Gene Expression and Radioiodine Uptake in Melanoma Cells: Novel Therapeutic Implications

**DOI:** 10.1371/journal.pone.0006200

**Published:** 2009-07-10

**Authors:** Peng Hou, Dingxie Liu, Meiju Ji, Zhi Liu, James M. Engles, Richard L. Wahl, Mingzhao Xing

**Affiliations:** 1 Division of Endocrinology and Metabolism, Department of Medicine, the Johns Hopkins University School of Medicine, Baltimore, Maryland, United States of America; 2 Division of Nuclear Medicine, Department of Radiology, the Johns Hopkins University School of Medicine, Baltimore, Maryland, United States of America; National Cancer Institute, United States of America

## Abstract

Both the MAP kinase and PI3K/Akt pathways play an important role in the pathogenesis of melanoma. We conducted the present study to test the hypothesis that targeting the two pathways to potently induce cell inhibition accompanied with thyroid iodide-handling gene expression for adjunct radioiodine ablation could be a novel effective therapeutic strategy for melanoma. We used specific shRNA approaches and inhibitors to individually or dually suppress the MAP kinase and PI3K/Akt pathways and examined the effects on a variety of molecular and cellular responses of melanoma cells that harbored activating genetic alterations in the two pathways. Suppression of the MAP kinase and PI3K/Akt pathways showed potent anti-melanoma cell effects, including the inhibition of cell proliferation, transformation and invasion, induction of G_0_/G_1_ cell cycle arrest and, when the two pathways were dually suppressed, cell apoptosis. Remarkably, suppression of the two pathways, particularly simultaneous suppression of them, also induced expression of genes that are normally expressed in the thyroid gland, such as the genes for sodium/iodide symporter and thyroid-stimulating hormone receptor. Melanoma cells were consequently conferred the ability to take up radioiodide. We conclude that dually targeting the MAP kinase and PI3K/Akt pathways for potent cell inhibition coupled with induction of thyroid gene expression for adjunct radioiodine ablation therapy may prove to be a novel and effective therapeutic strategy for melanoma.

## Introduction

Melanoma is a common skin cancer and recent decades have seen a markedly increase in its incidence worldwide [1]–[3]. In the United States alone, 62,480 new cases and 8,420 deaths from melanoma were estimated for the year of 2008 [3]. Although early-stage disease is curable through surgical excision, advanced metastatic melanoma is resistant to current treatments, with a rapidly progressive course and high mortality rate [4], [5]. A major effort in melanoma research has thus been to identify novel treatment strategies targeting major molecular pathways, particularly the Ras → Raf → MEK → MAP kinase/ERK (MAPK) and PI3K/Akt signaling pathways, which are commonly over-activated by genetic alterations, such as the *BRAF* mutations in the MAPK pathway [6] and the *PIK3CA* amplification and *PTEN* mutations in the PI3K/Akt pathway [7]–[9]. These two pathways play a fundamental role in the pathogenesis and progression of melanoma and are therefore important therapeutic targets for this cancer [10]–[15].

Radioiodine therapy based on the sodium/iodide symporter (NIS) gene transfer has been widely investigated as a potential therapeutic strategy for extrathyroidal malignancies [16]–[20]. NIS is normally expressed in the basal membrane of follicular thyroid cells, which transports iodide from blood stream into the cell for the biosynthesis of thyroid hormone [18], [21]. This process also involves several other key molecules, including thyroglobulin (Tg), which incorporates iodide through organification that involves thyroperoxidase (TPO). Thyroid transcription factor 1 (TTF1 or TITF1) and 2 (TTF2 or FOXE1) and PAX8 are involved in the regulation of these genes. Expression of many of these iodide-handling genes in the thyroid cell is up-regulated by the thyroid-stimulating hormone (TSH), which acts on the TSH receptor (TSHR) in the thyroid cell membrane. This is the molecular basis for the commonly used radioiodide ablation therapy for thyroid cancer, which is clinically facilitated by increasing the level of TSH in the blood of the patient either through thyroid hormone withdrawal or administration of recombinant human TSH [22], [23]. In papillary thyroid cancer (PTC), *BRAF* mutation (and hence activation of the MAPK pathway) was associated with decreased radioiodine avidity [24]–[26], which can be explained by *BRAF* mutation-associated silencing of thyroid iodide-handling genes, such as *NIS* [18], [27], *Tg* [27], and *TPO* [26]–[29]. Several previous studies also demonstrated involvement of the PI3K/Akt pathway in the regulation of thyroid iodide-handling genes. For example, expression of a mutant Ras that selectively stimulated the PI3K/Akt pathway markedly decreased TSH-induced NIS expression [30] and IGF-I could inhibit cAMP-induced NIS expression through activating the PI3K/Akt pathway in thyroid cells [31].

In recent clinical trials on various human cancers, including melanoma, targeting an individual pathway, such as the MAPK pathway or the PI3K/Akt pathway, or using a single agent generally failed to show significant clinical responses [9], [15], [32]. These results suggest that targeting multiple signaling pathways is a necessary therapeutic strategy for melanoma. Interestingly, a recent study showed common expression of TSHR in melanoma cells, but no or little expression in benign skin lesions [33], raising the possibility that other thyroid iodide-handling genes might also be expressible in melanoma cells. In the present study, we tested the therapeutic potential of dually targeting the MAPK and PI3K/Akt pathways in melanoma cells for synergistic/additive cell inhibition coupled with thyroid iodide-handling gene expression and radioiodide uptake as a novel therapeutic strategy for melanoma.

## Results

### Synergistic/additive inhibition of cell proliferation and induction of thyroid iodide-handling gene expression in melanoma cells by dually suppressing the MAPK and PI3K/Akt pathways using specific inhibitors

**Figure 1:**
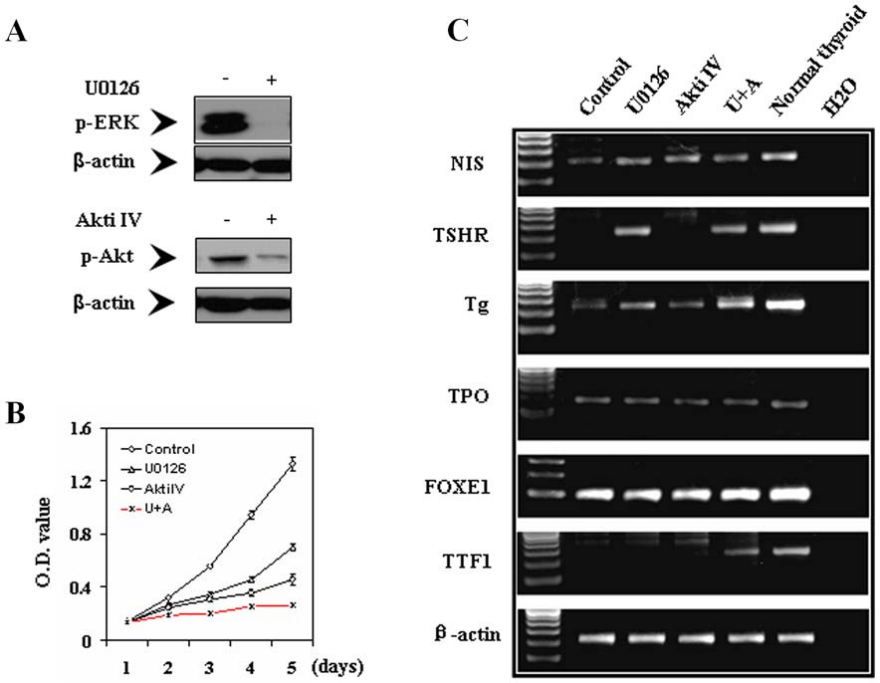
Effects of suppression of the MAPK and PI3K/Akt pathways by specific inhibitors on NPA cells. A) Inhibition of ERK and Akt phosphorylation by suppression of MAPK and PI3K/Akt pathways using the MEK-specific inhibitor U0126 and the Akt-specific inhibitor Akti IV, respectively. NPA cells were treated with U0126 at 10 µM, or with Akti IV at 0.5 µM for 30 h. DMSO was used as vehicle control. Cells were lysed for Western blotting assay. The activities of MAPK and PI3K/Akt pathways were reflected by the level of phosphorylated ERK and Akt detected with specific anti-phosphorylated ERK (p-ERK) and anti-phosphorylated Akt (p-Akt) antibodies. Immunoblotting with antibody against β-actin was used for quality control. B) Inhibition of cell proliferation by suppression of MAPK and PI3K/Akt pathways achieved with U0126 and Akti IV individually or in combination (U+A) as indicated. MTT assay was performed to evaluate cell proliferation over a 5-day course of treatment with U0126 and Akti IV. NPA cells were treated with 4 µM U0126 and 0.2 µM Akti IV, respectively – lower concentrations than those in A) were used for the two inhibitors in order to examine their additive/synergist effects. C) Expression of thyroid genes by dual suppression of MAPK and PI3K/Akt pathways using U0126 and Akti IV. For expression analysis of thyroid genes (*NIS*, *TSHR*, *Tg*, *TPO*, *FOXE1*, and *TTF1*), total RNA was isolated and RT-PCR was performed after cells were treated with U0126 and Akti IV individually or combination at the concentrations used in A) for 30 h.

We first used the melanoma cell line NPA cell, a cell clone derived from the melanoma cell line M14 cell [34], and inhibitors of the MAPK and PI3K/Akt pathways to test the therapeutic potential for melanoma of dully targeting the two pathways to induce potent cell inhibition coupled with thyroid iodide-handling gene expression for potential radioiodide treatment. As shown in Figure 1A, treatment of cells with the MEK inhibitor U0126 and the Akt inhibitor IV (Akti IV) [35] strongly inhibited phosphorylation of ERK (p-ERK) and Akt (p-Akt), respectively. Correspondingly, the two inhibitors inhibited cell proliferation partially when used individually and virtually completely when used in combination (Figure 1B). To explore the ability of suppressing the MAPK and PI3K/Akt pathways to induce the expression of iodide-metabolizing genes in melanoma cells, we tested the effects of U0126 and Akti IV on NPA cells which had no or low basal expression of iodide-handling genes. As shown in Figure 1C, expression of several iodide-handling genes, including *NIS*, *TSHR*, *Tg*, and *TTF1*, was induced or dramatically enhanced after treatment of cells with U0126 or Akti IV, particularly U0126. Similar effects of these inhibitors of the MAPK and PI3K/Akt pathways on cell inhibition and iodide-handling gene expression were seen in DRO cells (data not shown). This phenomenon of induced expression of thyroid genes in melanoma cells was further investigated using the real-time quantitative RT-PCR approach in subsequent studies as described below.

### Expression of iodide-handling genes in various melanoma cell lines induced by suppressing the MAPK and PI3K/Akt pathways and its enhancement or synergy by TSH stimulation

**Figure 2:**
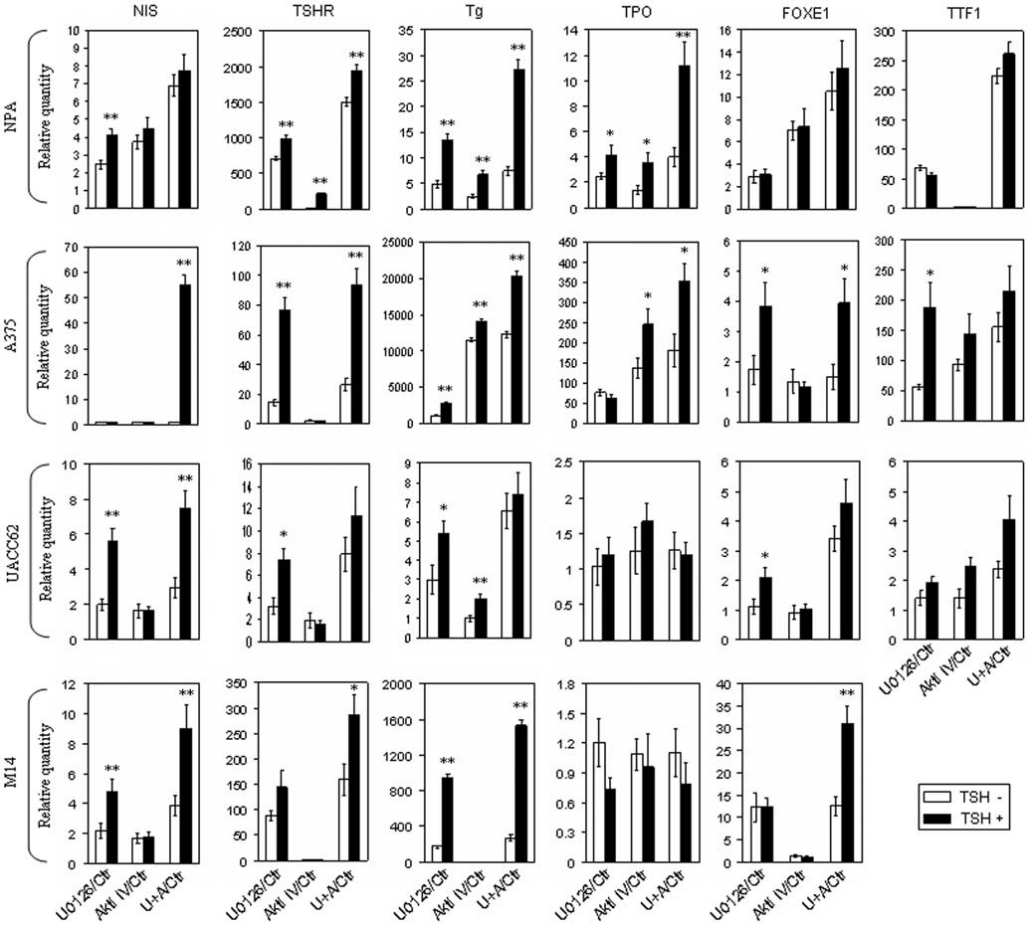
Effect of TSH stimulation on expression of thyroid genes mediated by suppressing MAPK and PI3K/Akt pathways, individually or in combination, in melanoma cells. Melanoma cells, as indicated, were treated with specific inhibitors (U0126 and Akti IV) as described in Figure 1. Before RNA was extracted, cells were treated with 40 mU/ml TSHβ for 6 h. Details are described in the Materials and Methods. Data are presented as the mean±SD of values from three assays. In comparison with control, *, *P*<0.05; **, *P*<0.01.

Encouraged by the novel finding of the expression of iodide-handling genes upon suppression of the MAPK and PI3K/Akt pathways in the NPA cell, we extended this study to other melanoma cell lines. As shown in Figure 2, in several melanoma cell lines tested, including M14, UACC62, A-375 and, again, NPA cells, dually suppressing the MAPK and PI3K/Akt pathways by U0126 and Akti IV showed synergistic/additive effects on the expression of most of the iodide-handling genes compared with suppressing either pathway alone. Since TSHR plays an important role in up-regulating the iodide-handling genes in thyroid cells [21] and is expressed in melanoma cells upon suppression of the MAPK and PI3K/Akt pathways, we investigated whether TSH treatment could affect the expression of iodide-handling genes in melanoma cells. Remarkably, treatment of melanoma cells with TSH enhanced or synergized the expression of many of the iodide-handling genes induced either by suppression of one signaling pathway alone or simultaneous suppression of both pathways (Figure 2). This effect of TSH seemed to be most commonly evident when the MAPK pathway was suppressed.

### Cellular inhibition of melanoma cells by siRNA knockdown of BRAF and Akt-1/2

**Figure 3:**
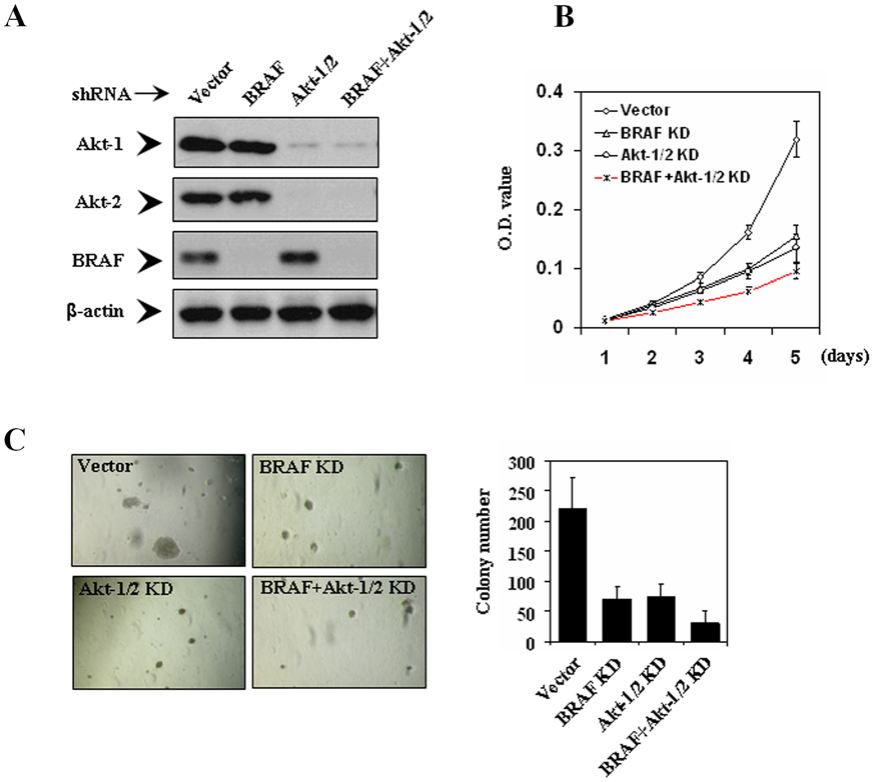
Effects of stable siRNA knockdown of BRAF and Akt-1/2, individually or dually, on cell proliferation and colony formation. A) NPA cells were infected with lentivirus expressing BRAF or Akt-1/2 siRNAs or both and stable populations were selected with 2 µg/ml puromycin. The empty vector was used as the control. After a 2-week selection, cells were lysed and immunoblotted with BRAF, Akt-1 and Akt-2 antibodies. The antibody against β-actin was used for quality control of Western blotting. B) Proliferation rate of NPA cells stably transfected with various siRNA constructs as described in A) was measured with MTT assay daily over a 5-day course. Results are expressed as means±SD of three independent experiments. C) Representative results of colony formation in soft agar of thyroid cancer cells with stable transfections with different siRNA constructs, including empty vector (vector), BRAF siRNA (BRAF KD), Akt-1/2 siRNA (Akt-1/2 KD), and combination of Akt-1/2 and BRAF siRNA (BRAF+Akt-1/2 KD). Data represent means±SD of three independent experiments. KD, knockdown (same as in all other Figures).

**Figure 4:**
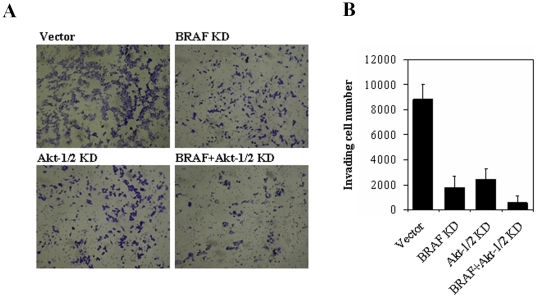
Effects of stable siRNA knockdown of BRAF and Akt-1/2, individually or dually, on the invasion of NPA cells. Invading rate of cells stably transfected with specific siRNAs constructs to knock down BRAF and Akt-1/2, individually or dually, as described in Figure 3 was measured using Matrigel-coated transwell cell culture chambers. A) Shown are representative results of invasive NPA cells. B) The bar graphs, corresponding to (A), show means±SD of the numbers of invading cells from three independent experiments.

The above findings from the experiments using pharmacological inhibitors strongly suggest that dually targeting the MAPK and PI3K/Akt signaling pathways to induce both cellular inhibition and thyroid gene expression for adjunct radioiodine ablation therapy could be a novel and effective therapeutic strategy for melanoma. To more specifically test this hypothesis, in the next series of experiments, we used siRNA approaches to specifically knock down the MAPK and PI3K/Akt pathways, individually or dually in a selected melanoma cell line, the NPA cell. We pursued stable transfection with dual knockdown of Akt-1 and Akt-2 in combination with BRAF knockdown to achieve dual suppression of the two signaling pathways. To this end, we stably transfected NPA cells with pSicoR-PGK-puro encoding specific shRNA homologue sequences for Akt-1 and Akt-2 (Akt-1/2), superimposed with or without stable transfection of specific BRAF siRNA. Cells stably infected with lentivirus expressing BRAF and Akt-1/2 siRNA were successfully selected using puromycin, as demonstrated by effective suppression of expression of the corresponding proteins. Specifically, as shown in Figure 3A, BRAF and Akt-1/2 siRNA stably and virtually completely inhibited the expression of BRAF and Akt-1/2, respectively, compared with empty vectors. As shown in Figure 3B and C, specific BRAF siRNA strongly inhibited proliferation and transformation of cells, the latter being reflected by anchorage-independent colony formation on soft agar. Similarly, specific Akt-1/2 siRNA also exhibited dramatic inhibition of cell proliferation and transformation (Figure 3B and C). Further inhibition of cell proliferation and transformation was achieved by dual knockdown of BRAF and Akt-1/2 (Figure 3B and C). Migration/invasion of the NPA cells on Matrigel was also inhibited by stable siRNA knockdown of either pathway, with further inhibition achieved with dual knockdown of the two pathways (Figure 4).

### Induction of G_0_/G_1_ cell cycle arrest and apoptosis by siRNA knockdown of BRAF and Akt-1/2

**Figure 5:**
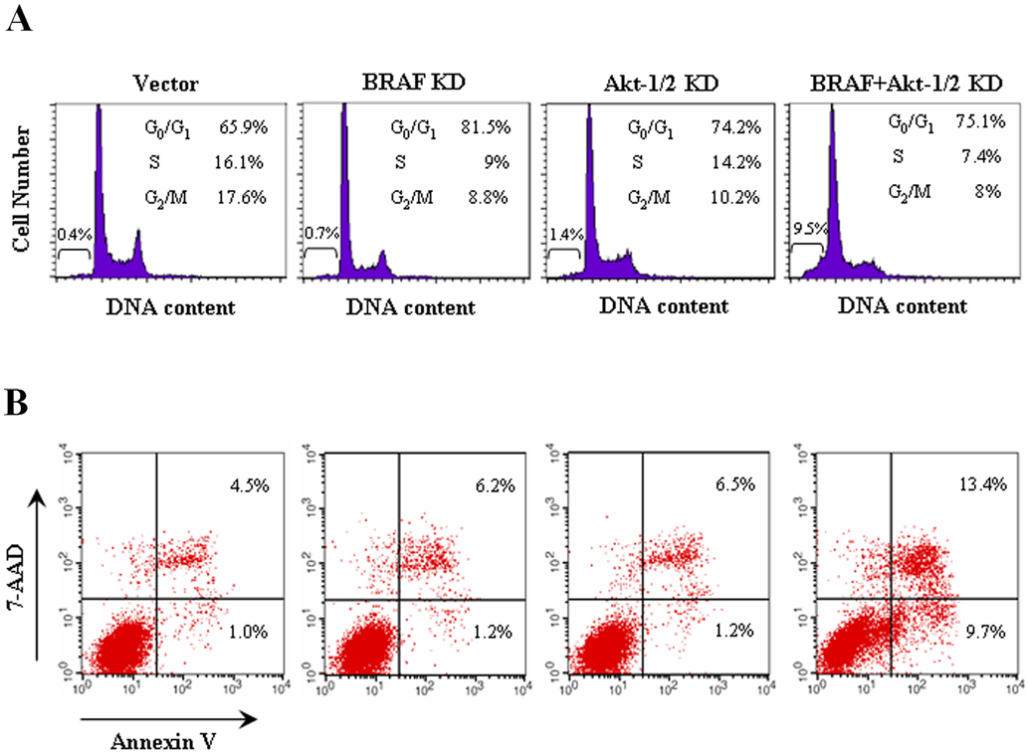
Effects of siRNA knockdown of BRAF and Akt-1/2, individually or dually, on G_0_/G_1_ cell cycle arrest and apoptosis of NPA cells. NPA cells were transiently transfected with specific siRNAs to knock down BRAF and Akt-1/2, individually or dually as indicated. After a 4-day culture, DNA content (A) was measured by flow cytometry to determine cell cycle fractions. The fraction of apoptotic cells (sub-G_0_) is indicated (A). Cell apoptosis (B) was measured by Annexin V staining and flow cytometry. The right lower quadrant of each plot contains early apoptotic cells, whereas the right upper quadrant contains late apoptotic cells. Details are described in the Materials and Methods.

To investigate further the cellular events involved in the inhibition of cell proliferation by dual suppression of MAPK and PI3K/Akt pathways, we transiently transfected BRAF and Akt-1/2 siRNA in NPA cells and measured cell cycle and apoptotic patterns by flow cytometric analysis of DNA content and Annexin V expression (Figure 5). Compared with control vector, siRNA knockdown of BRAF and Akt-1/2 caused an increase in the G_0_/G_1_ fraction from 62.8±2.4% to 78.8±2.2% (*P*<0.01) and to 71±2.4% (*P*<0.05), respectively (Figure 5A). With dual knockdown of BRAF and Akt-1/2, there was a dramatic synergistic increase in the sub-G_0_ fraction in comparison with control vector (8.1±1.1% vs. 0.6±0.2%, *P*<0.001) or knockdown of BRAF (8.1±1.1% vs. 0.8±0.2%, *P*<0.001) or Akt-1/2 (8.1±1.1% vs. 1.1±0.3%, *P*<0.001) alone, reflecting increased cell apoptosis, and the G_0_/G_1_ fraction was correspondingly only moderately increased (Figure 5A). These data were consistent with the apoptotic patterns shown in Figure 5B, which showed a dramatic increase in both early and late apoptosis with dual knockdown of BRAF and Akt-1/2 in comparison with control vector (8.5±0.9% vs. 1.1±0.4%, *P*<0.001; 11.9±1.4% vs. 3.8±0.6%, *P*<0.01), respectively. In contrast, knockdown of BRAF or Akt-1/2 alone had a much smaller effect on cell apoptosis. These data suggest that both increased G_0_/G_1_ cell cycle arrest and cell apoptosis were involved in the inhibition of NPA cell growth by suppression of MAPK and PI3K/Akt pathways. The effects were most significant with dual knockdown of the MAPK and PI3K/Akt pathways, particularly for cell apoptosis.

### NIS protein expression and radioiodide uptake in melanoma cells induced by suppressing the MAPK and PI3K/Akt pathways

**Figure 6:**
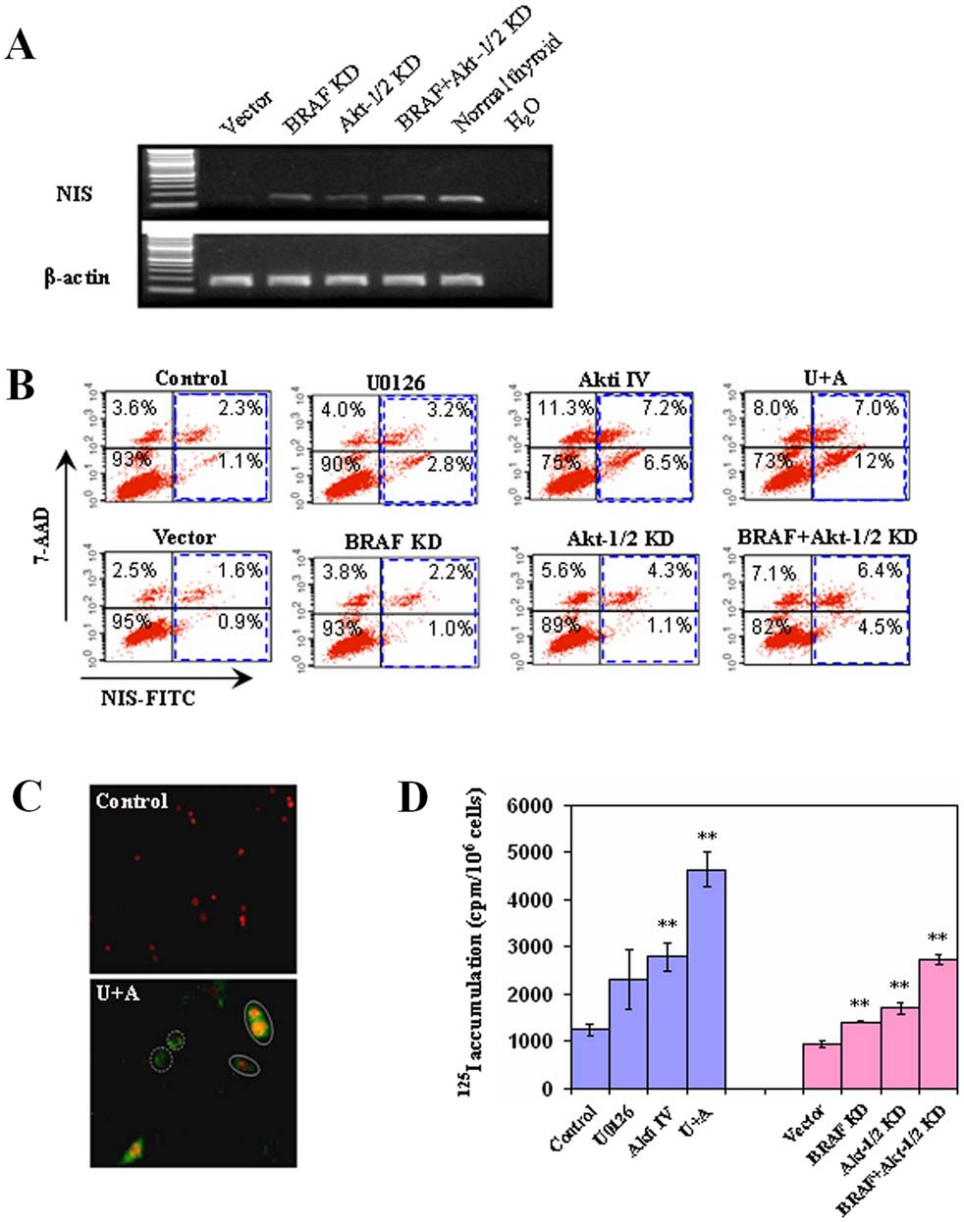
Effects of suppression of MAPK and PI3K/Akt pathways, individually or in combination, on the expression of *NIS* and radioiodine uptake in NPA cells. A) NPA cells were stably transfected with specific siRNAs to knock down BRAF and Akt-1/2, individually or dually as described in Figure 3. After a serum starvation for 24 h, total RNA was isolated for expression analysis for the indicated thyroid iodide-metabolizing genes. RT-PCR analysis was performed for the expression of *NIS* in NPA cells. B) Flow cytometric measurement of NIS protein expression. NPA cells were treated with specific inhibitors (upper panel) as described in Figure 1 or stably transfected with specific siRNAs to knock down BRAF and Akt-1/2, individually or in combination as described in Figure 3 (lower panel). NIS protein levels were measured by flow cytometry. The blue frames indicate the cells expressing NIS protein, including dead (right upper quadrant; positive for both 7-AAD and NIS) and living (right lower quadrant, positive only for NIS) cells. C) Immunofluorescent localization of NIS. After a 30-h combined treatment with both U0126 and Akti IV (as in B), cells were analyzed by immunofluorescent microscopy using anti-NIS and FITC-coupled secondary antibody and double immunofluorescence with the red color representing 7-AAD nuclear staining and the green color representing NIS expression and localization. Cells marked with dash circles are intact living cells that do not have 7-AAD nuclear staining. NIS staining in these cells represents NIS protein expression exclusively on the cell membrane. Cells marked with solid circles show double colors, suggesting that the cells were not intact and therefore both cell membrane NIS staining and 7-AAD nuclear staining occurred. The NIS expression is in striking contrast with the control cells which did not show any NIS staining even in the broken cells that showed nuclear staining (red color) with 7-ADD. D) *In vitro* radioiodide uptake. NPA cells were treated with specific inhibitors or stably transfected with various siRNA constructs as indicated. Cells were subsequently incubated with 1 µCi ^125^I/0.5 ml/well on 12-well plates for 1 h. Cells were then washed and harvested for radioactivity measurement using a gamma-counter as described in the Materials and Methods. Data are expressed as the mean±SD of values from three assays. **, *P*<0.01, compared with control or empty vector.

As NIS plays the most important role in cellular iodide uptake, we next explored further the effect of suppressing the MAPK and PI3K/Akt pathways on NIS expression in melanoma cells. As shown in Figure 6A, stable knockdown of BRAF and/or Akt-1/2 with specific siRNA induced the expression of NIS in NPA cells, consistent with the results achieved with treatments of cells with pharmacological inhibitors (Figure 1C and Figure 2). We next investigated the effects of suppressing the MAPK and PI3K/Akt pathways on the expression of NIS protein in NPA cells. As shown in Figure 6B, U0126 and Akti IV increased the NIS expression from 1.1+2.3% to 2.8+3.2% and 6.5+7.2%, respectively, and to 12+7.0% with combination of the two (Figure 6B, upper panel). Although both U0126 and Akti IV induced NIS protein expression, Akti IV showed a more pronounced effect (Figure 6B, upper panel). Combination of U0126 and Akti IV showed significantly enhanced or synergized effects on NIS protein expression (Figure 6B, upper panel). Similarly, siRNA knockdown of BRAF and Akt-1/2 could each increase the NIS protein expression, albeit modestly, and dual knockdown of BRAF and Akt-1/2 showed enhanced effects (Figure 6B, lower panel). We also performed immunofluorescent microscopy, which showed that dual inhibition of the MAPK and PI3K/Akt pathways by U0126 and Akti IV robustly induced the expression of NIS protein on cell membranes (Figure 6C).

Given the induction of expression of iodide-handling genes in melanoma cells by suppression of the MAPK and PI3K/Akt pathways, we finally examined functionally the ability of these cells to take up radioiodide. As shown in Figure 6D, treatment of NPA cells with U0126 or Akti IV significantly increased radioiodide uptake and combination of the two inhibitors resulted in an enhanced uptake. Stable knockdown of BRAF or Akt-1/2 could also increase radioiodide uptake, which was enhanced by simultaneous knockdown of the two.

## Discussion

Recent advances in understanding aberrant signaling of molecular pathways in melanoma provide the bases for the current development of novel therapies for this cancer. Of particular importance are the MAPK and PI3K/Akt pathways, which, through their genetic alterations, play a critical role in the development and pathogenesis of melanoma and are therefore tested vigorously as important therapeutic targets for this cancer [6], [10]–[15]. Several clinical trials targeting these pathways in various cancers, including melanoma, have been recently completed [9], [15], [32]. Disappointingly, however, little or no anti-tumor response has been achieved in these trials, which were single-agent based and, hence, targeted limited pathways. It becomes increasingly questionable whether targeting only single or limited pathways is an effective therapeutic strategy for cancers [15], [32]. Given the importance of both the MAPK and PI3K/Akt pathways in melanoma, simultaneously targeting the two pathways, instead of either alone, may prove to be a particularly effective therapeutic strategy for this cancer. In the present study, we tested this hypothesis by dually and specifically suppressing the MAP kinase and PI3K/Akt pathways in melanoma cells and examining molecular and cellular consequences. Our main theme in this effort was to particularly test a novel therapeutic strategy of inducing both potent cellular inhibition and thyroid iodide-handling gene expression for potential adjunct radioiodide therapy for melanoma.

In addition to using specific inhibitors of the pathways, we used shRNA approaches to simultaneously and specifically knock down Akt-1/2 and BRAF, resulting in synergistic/additive inhibition of melanoma cell proliferation, colony formation and invasion, as well as apoptosis. Due to the technical complexities and laborious efforts required in shRNA approaches for simultaneous knockdown of multiple signaling pathways, previous studies on certain cancer cell lines, including one in melanoma cells [14], only attempted using drug inhibitors for simultaneous suppression of the MAPK and PI3K/Akt pathways. The present study represents the first to use the shRNA approach to simultaneously and specifically knock down the MAPK and PI3K/Akt signaling in cancer cells, definitely demonstrating the therapeutic potential of dually targeting the two pathways for cancers, such as melanoma. Of particular note in the present study is that although suppression of either the MAPK or PI3K/Akt pathway could cause significant inhibition of melanoma cell proliferation, suppression of either alone caused little cell apoptosis. Previous studies targeting single pathways using single agents also only showed cell cycle arrest at the G_0_/G_1_ phase, but not apoptosis, in certain cancer cells [36], [37]. These results are consistent with the recent clinical trials on melanoma using single agents that showed little anti-tumor effect other than only tumor stability in some cases [9], [15], [32]. Remarkably, the present study showed that combined suppression of the MAPK and PI3K/Akt pathways synergistically promoted cell apoptosis, suggesting that both pathways need to be removed to induce melanoma cell death. This result is consistent with an interesting previous observation that either pathway was sufficient to protect melanoma cells from anoikis, a type of apoptosis induced by loss of normal cell contact [38]. Thus, therapeutic approaches dually targeting the MAPK and PI3K/Akt pathways are likely to be more effective in killing melanoma cells harboring genetic alterations that activate both pathways. Consequently, it may be expected that a clinical trial using combined agents targeting both the MAPK and PI3K/Akt pathways would likely show significant anti-melanoma effectiveness, unlike the recently completed single agent-based clinical trials [9], [15], [32]. Although apoptosis was induced in a large portion of melanoma cells by simultaneously targeting MAPK and PI3K/Akt pathways, it did not seem to occur in all cells, suggesting that some alternative pathways may also be involved.

Radioiodine ablation therapy is an effective and standard treatment for thyroid cancer, which is routinely administered after thyroidectomy in most thyroid cancer patients [22], [23], [39], [40]. This treatment takes advantage of the unique iodide-handling machinery in thyroid cells, involving several key molecules, such as NIS, TSHR, TPO, Tg, and several thyroid transcription factors. These genes are frequently silenced in thyroid cancer, particularly in association with aberrant activation of the MAPK and PI3K/Akt pathways [18], [26]–[29], [41]. Suppression of the MAPK and PI3K/Akt pathways could partially restore the expression of thyroid iodide-handling genes in thyroid cancer cells [36, and Hou and Xing, unpublished data]. A previous study interestingly showed that melanoma cells also expressed TSHR [33]. Based on these data, we suspected that melanoma cells might have the ability to express other thyroid iodide-handling genes that were regulated by the MAPK and PI3K/Akt pathways. This was proven to be the case in the present study. In fact, we demonstrated that suppression of either of the two pathways could induce the expression of some thyroid iodide-handling genes and dual suppression of the two pathways had a synergistic/additive effect on their expression in melanoma cells. As an example, NIS, the most important molecule involved in thyroid cellular uptake of iodide, was robustly expressed in the cell membrane with dual suppression of the MAPK and PI3K/Akt pathways either using inhibitors or specific shRNA approaches. As in normal thyroid cells, TSH significantly enhanced the expression of these genes induced by suppression of the MAPK and PI3K/Akt pathways in melanoma cells. Importantly, we also demonstrated that expression of thyroid iodide-handling genes effectively conferred melanoma cells the ability to take up radioiodide. These results have important novel therapeutic implications for melanoma: radioiodide ablation, as in thyroid cancer, might be therapeutically effective for melanoma in conjunction with the use of agents to inhibit the MAPK and PI3K/Akt pathways. For radioiodide treatment of thyroid cancer, TSH is routinely raised either by thyroxine withdrawal or administration of human recombinant TSH to enhance radioiodide uptake and ablation of thyroid cancer cells [21], [23], [39], [40]. This strategy could be similarly used for melanoma given the expression of TSHR induced by suppressing the MAPK and PI3K/Akt pathways and the enhancement of expression of other iodide-metabolizing genes by TSH in melanoma cells demonstrated in the present study. In recent years, NIS gene transfer therapy to confer non-thyroid cancers the sensitivity to radioiodide ablation therapy has been widely investigated as a potential therapeutic strategy for human cancers [18], [42], [43]. Yet, the technical complexities, inadequate therapeutic efficiencies, and other issues associated with NIS gene transfer have so far prevented it from rapid and successful clinical use. Our demonstration of the induced expression of thyroid iodide-handling genes and radioiodide uptake in melanoma cells opened the possibility for a potentially safe, effective, and easy alternative approach to therapeutic use of radioiodide in melanoma. Given the results in the present study, it is attractive to propose clinical trials to test the novel therapeutic strategy of simultaneously targeting the MAPK and PI3K/Akt pathways for both synergistic/additive cellular inhibition and thyroid gene expression for adjunct radioiodide treatment in melanoma. Such clinical trials are feasible particularly given the current availability of several safe and potent inhibitors of the MAPK and PI3K/Akt pathways, such as the MEK and Akt inhibitors [9], [15], [32].

## Materials and Methods

### Human melanoma cell lines

The melanoma cell line UACC62 was obtained from the National Cancer Institute (NCI); melanoma cells lines M14 and A375 were obtained from American Type Culture Collection (ATCC). The NPA cell and DRO cell were provided by Dr. Guy J.F. Juillard (University of California-Los Angeles School of Medicine, Los Angeles, CA). These two cell lines were previously mistakenly labeled as thyroid cancer cell lines [44] and have now been demonstrated to be clones derived from the melanoma cell lines M14 and A375, respectively [34]. NPA, M14 and UACC62 cells harbor genetic alterations that can activate both the MAPK and PI3K/Akt pathways: *BRAF* V600E mutation and *PIK3CA* amplifications in NPA and M14 cells, and *BRAF* V600E mutation and inactivating *PTEN* mutations (homozygous) in UACC62 cells. A375 and DRO cells harbor *BRAF* V600E mutation. Cells were cultured as previously described [44], [45]. Cells were treated with the MEK inhibitor U0126 (Sigma) at 10 µM or Akt inhibitor IV (Akti IV, Calbiochem) at 0.5 µM for 30 h. DMSO was used as the vehicle control. In some experiments as indicated, cells were additionally treated with bovine TSH (TSHβ) (Sigma) to test its effect on gene expression.

### Western blotting assay

Cells were lysed in Radio Immuno Precipitation Assay (RIPA) buffer. Cellular proteins were resolved by electrophoresis on a SDS-polyacrylamide (10%) gel (SDS-PAGE), transferred onto Polyvinylidene Fluoride (PVDF) membranes (Amersham Pharmacia Biotech, Piscataway, NJ), and immunoblotted with specific primary antibodies. Anti-BRAF (Sc-166), anti-phospho-AKT (Sc-7985-R), anti-phospho-ERK (Sc-7383), and anti-Actin (Sc-1616-R) were purchased from Santa Cruz (Santa Cruz, CA). Anti-Akt-1 (#2967) and anti-Akt-2 (#2964) were purchased from Cell Signaling Technologies, Inc. (Beverly, MA). Antigen-antibody complexes were visualized using HRP-conjugated anti-mouse (Sc-2005, Santa Cruz, CA) or anti-rabbit (Sc-2004, Santa Cruz, CA) IgG antibodies and ECL Western Blotting Analysis System (Amersham Pharmacia).

### RNA extraction, RT-PCR analysis, and real-time quantitative RT-PCR analysis

Total RNA was isolated using TRIzol reagent according to the instructions of the manufacturer (Invitrogen). Normal human thyroid RNA samples purchased from Stratagene (La Jolla, CA) were used as a positive control. RNA samples were quantified using Multiskan Spectrum (Thermo, Electron Corporation) prior to use. The A260/A280 ratios were between 1.8 and 2.0. Two µg of total RNA was converted to cDNA on an iCycler Thermal Cycler (Bio-Rad) using Oligo-dT and SuperScript II according to the instructions of the manufacturer (SuperScript First-Strand Synthesis kit, Invitrogen). Conventional RT-PCR amplification was carried out to amplify *NIS*, *TSHR*, *Tg*, *TPO*, *FOXE1*, and *TTF1*. The *â-actin* gene was run in parallel for quality control. The primer sequences were presented in Supplementary Table S1 and 0.4 µM each primer was used in each reaction. PCR products were resolved by 1.5% agarose gel electrophoresis and visualized by ethidium bromide staining. Real-time quantitative RT-PCR analysis was performed to evaluate expression of thyroid genes on an ABI Prism 7900HT Sequence Detector (Applied Biosystems), using SYBR GreenER qPCR SuperMix according to the instructions of the manufacturer (Applied Biosystems). This reaction mixture contained AmpErase (UNG). The expression value of each gene was normalized to â-actin cDNA to calculate the relative amount of RNA present in each sample according to the 2^−ΔΔCt^ method [46]. *β-actin* is a commonly used reference gene for the type of analysis we performed. We also tested other two frequently used reference genes, *GAPDH* and *HPRT1*, in some experiments and found similar results for the expression of thyroid genes, suggesting that *β-actin* is a valid normalizer in the present study. Each sample was run in triplicate. The primers of intron-spanning thyroid-specific genes, *NIS*, *TSHR*, *Tg*, *TPO*, *FOXE1*, and *TTF1* were designed using Primer Express (Applied Biosystems, CA) following the recommended guidelines based on sequences from GenBank. The primer sequences are presented in Supplementary Table S2 and 0.25 µM each primer was used in the reaction. The specificity of real-time quantitative PCR for all these genes was confirmed by running the PCR products on a 1.5% agarose gel to show single specific bands of the PCR products at the expected sizes (Supplementary Figure S1), followed by direct sequencing of the PCR products using Big Dye terminator V 3.0 cycle sequencing reagents (Applied Biosystems, Foster City, CA). The sequences of the real-time quantitative PCR products were confirmed to represent the correct genes (Supplementary Figure S2). The reliability of the real-time quantitative PCR was also assured by the nearly perfect standard curves established using serial dilutions of normal human thyroid cDNA for all these genes (Supplementary Figure S3). The copy number of cDNA analyzed using this real-time quantitative PCR technique for each gene was therefore reliable in the present study.

### Lentivirus-mediated RNA interference of BRAF, Akt-1, and Akt-2

The lentiviral pSicoR-PGK-puro vectors (Addgene Inc. Cambridge, MA, USA) encoding hairpin RNA sequences were used to knock-down BRAF and specific Akt isoforms. The hairpin sequences used for BRAF and combined Akt-1 and Akt-2 (Akt-1/2) were presented in Supplementary Table S3. To generate lentiviral particles, human embryonic kidey 293 cells (ATCC, Manassas, VA, USA) were co-transfected with the lentiviral vector and compatible packaging plasmid mixture using Lipofectamine 2000 (Invitrogen), following the manufacturer's instructions. Melanoma cells were exposed to lentivirus-containing supernatant for 16 hours in the presence of Polybrene (Sigma). After 3–4 days, the cells were serum-starved (0.5% FBS) and harvested 24 hours later in RIPA lysis buffer (Santa Cruz, CA). Western blotting assays were used to detect the protein expression of BRAF, Akt-1, and Akt-2.

### Cell proliferation assay

Cells (800/well) were seeded into 96-well plates and cultured with 2.5% FBS. MTT [3-(4,5-Dimethylthiazol-2-yl)-2,5-Diphenyltetrazolium Bromide] assay was performed daily over a 5-day time course to evaluate cell numbers using a MTT cell proliferation assay kit (ATCC, Manassas, VA, USA) following the manufacturer's instructions.

### Colony formation assay

For soft-agar colony-formation assay, 1×10^5^ cells were plated into 6-well plates with a bottom layer of 0.6% agar and a top layer of 0.3% agar. Following the hardening of soft agar, plates were incubated at 37°C with 5% CO_2_. After 2–3 weeks of culture, colonies were counted and photographed automatically by an AlphaImager Imaging System (Alpha Innotech).

### Cell invasion assay

Cell invasion was assayed in triplicates using Matrigel-coated Transwell cell culture chambers (#354481, BD Biosciences). Briefly, cells (1.5×10^5^ cells/well) suspended in serum-free medium were placed in the upper chamber of the Transwell insert, and RPMI 1640 medium containing 10% FBS was added to the lower chamber. Following a 24 h-incubation at 37°C with 5% CO_2_, non-invasive cells in the upper chamber were removed and invasive cells were fixed in 100% methanol and stained with 0.5% crystal violet in 2% ethanol. The numbers of invasive cells were automatically counted and photographed by an AlphaImager Imaging System (Alpha Innotech).

### Cell cycle analysis

Cells were harvested, washed twice in PBS, and resuspended in 70% ethanol on ice for at least 30 min. After centrifugation, 1×10^6^ cells transiently transected with various siRNA constructs were resuspended in 1 ml of propidium iodide staining solution (50 µg of propidium iodide, 1 mg of RNase A, and 1 mg of glucose per 1 ml PBS) and incubated at room temperature for 30 min. Cell cycles were analyzed based on DNA contents by FACS using a LSR Flow Cytometer (BD Biosciences, NJ).

### Apoptosis assay

Cells were transiently transfected with various siRNA constructs. After a 24-h serum starvation, cells were harvested, washed with PBS, and subjected to sequential staining with Annexin V-PE Apoptosis Detection Kit (BD Biosciences) by two-color flow cytometry, according to the manufacturer's protocol. Cells that were Annexin V-positive and 7-AAD-negative served as early apoptotic population. Cells that were both Annexin V- and 7-AAD-positive served as late apoptotic population.

### Flow cytometry analysis of NIS expression

Cells treated with specific inhibitors and various siRNA constructs to induce the expression of NIS were incubated with VJ2 α-hNIS mAb (a gift from Dr. Sabine Costagliola at the Free University of Brussels) [47] diluted at 1∶20 in FACS buffer (3% FBS, 0.02% NaN_3_ in PBS) at 4°C for 1 h. Cells were then washed once with FACS buffer and incubated with FITC-conjugated α-mouse IgG (Sigma) diluted at 1∶100 in FACS buffer at 4°C for 1 h. Cells were washed again in FACS buffer, resuspended in 2 ml of FACS buffer with 7-AAD, and analyzed by FACS using a LSR Flow Cytometer (BD Biosciences, NJ). Secondary antibody alone was used as a negative control. Fluorescent microscopic examination was conducted to monitor NIS expression (Nikon Corporation, Tokyo, Japan).

### Radioactive iodine uptake assay

Cells (1×10^6^ cells/well) treated under the indicated conditions were seeded in 12-well plates. Cells in 0.5 ml/well were incubated with RPMI 1640 medium containing 1 µCi Na^125^I and 5 µM non-radioactive NaI for 1 h at 37°C with 5% CO_2_. The medium was subsequently aspirated and cells were quickly washed twice with ice-cold Hank's balanced salt solution (HBSS) and harvested with tripsin-EDTA. Cells were collected and radioactivity was counted by a gamma-counter.

### Statistical analysis

All the experiments were similarly done at least three times. Most of the measurements were performed in triplicate and some in duplicates. The statistical significance of differences between two groups of data was analyzed by paired *t*-test and a *P* value of<0.05 was considered significant. Unless indicated, the results shown in the figures are representatives.

## Supporting Information

Table S1 Primer sequences used in RT-PCR analysis of the expression of thyroid iodide-handling genes
(0.04 MB DOC)

Table S2 Primer sequences used for quantitative RT-PCR analysis of the expression of thyroid iodide-handling genes
(0.04 MB DOC)

Table S3 Hairpin RNA sequences used to specifically knock down BRAF, Akt-1 and -2
(0.03 MB DOC)

Figure S1 Specificity analysis of real-time quantitative PCR for all investigated genes by running gel. M, DNA Marker.
(0.15 MB TIF)

Figure S2 Specificity analysis of real-time quantitative PCR for all investigated genes by sequencing.
(1.48 MB TIF)

Figure S3 Efficiency evaluation of real-time quantitative PCR for all investigated genes by standard curves.
(1.32 MB TIF)
