# Supplementary material for: Induction of Thyroid Gene Expression and Radioiodine Uptake in Melanoma Cells: Novel Therapeutic Implications
Source: PLoS One. 2009 Jul 10;4(7):e6200. doi: 10.1371/journal.pone.0006200 (PMC2703805; doi:10.1371/journal.pone.0006200)
Supplement: Table S2 — Primer sequences used for quantitative RT-PCR analysis of the expression of thyroid iodide-handling genes (0.04 MB DOC) [file pone.0006200.s002.doc]

**Table S2**. Primer sequences used for quantitative RT-PCR analysis of the expression of thyroid iodide-handling genes

| **Genes** | **Forward primer (5’→3’)** | **Reverse primer (5’→3’)** | **Product length** | **Annealing Temp.** |
| --- | --- | --- | --- | --- |
| *NIS* ([NM_000453](http://www.ncbi.nlm.nih.gov/entrez/query.fcgi?cmd=Search&db=Nucleotide&term=NM_000453)) | CCTGCTAACGACTCCAGCA | CCAGGGCACCGTAATAGAGA | 106bp | 60C |
| *TSHR* ([NM_000369](http://www.ncbi.nlm.nih.gov/entrez/query.fcgi?cmd=Search&db=Nucleotide&term=NM_000369)) | GATATTCAACGCATCCCCAG | AGCTGCTGCAGAGTCACATC | 149bp | 60C |
| *Tg* ([NM_003235](http://www.ncbi.nlm.nih.gov/entrez/query.fcgi?cmd=Search&db=Nucleotide&term=NM_003235)) | CACCAACTCCCAACTTTTCC | CAACTGACCTCCTTTGCCA | 123bp | 60C |
| *TPO* (NM_000547) | ACTTGGATCTCCATGTCGCT | GCAGTGTGGATTTAGTGCCA | 106bp | 60C |
| *FOXE1* ([NM_004473](http://www.ncbi.nlm.nih.gov/entrez/query.fcgi?cmd=Search&db=Nucleotide&term=NM_004473)) | GCTGGTTTTCCCTGTCTCTG | AGATGGGGGAGACTGAAGGT | 100bp | 60C |
| *TTF1* ([NM_003317](http://www.ncbi.nlm.nih.gov/entrez/query.fcgi?cmd=Search&db=Nucleotide&term=NM_003317)) | ACCAGGACACCATGAGGAAC | GCTCATGTTCATGCCGCT | 116bp | 60C |
| *ß-Actin* ([NM_001101](http://www.ncbi.nlm.nih.gov/entrez/query.fcgi?cmd=Search&db=Nucleotide&term=NM_001101)) | GCACAGAGCCTCGCCTT | GTTGTCGACGACGAGCG | 93bp | 60C |
