# Supplementary material for: Induction of Thyroid Gene Expression and Radioiodine Uptake in Melanoma Cells: Novel Therapeutic Implications
Source: PLoS One. 2009 Jul 10;4(7):e6200. doi: 10.1371/journal.pone.0006200 (PMC2703805; doi:10.1371/journal.pone.0006200)
Supplement: Table S3 — Hairpin RNA sequences used to specifically knock down BRAF, Akt-1 and -2 (0.03 MB DOC) [file pone.0006200.s003.doc]

**Table S3.** Hairpin RNA sequences used to specifically knock down BRAF, Akt-1 and -2

| **Genes** | **Forward sequence (5’→3’)** | **Reverse sequence (5’→3’)** |
| --- | --- | --- |
| *BRAF* | TGCATCAATGGATACCGTTAT TCAAGAGATAACGGTATCCAT TGATGCTTTTTTC | TCGAGAAAAAAGCATCAATGGATA CCGTTATCTCTTGAATAACGGTAT CCATTGATGCA |
| *Akt-1/2* | TGTGGTCATGTACGAGATGAT TCAAGAGATCATCTCGTACAT GACCACTTTTTTC | TCGAGAAAAAAGTGGTCATGTACG AGATGATCTCTTGAATCATCTCGT ACATGACCACA |
