# Supplementary figures and images for: Induction of Thyroid Gene Expression and Radioiodine Uptake in Melanoma Cells: Novel Therapeutic Implications
Source: PLoS One. 2009 Jul 10;4(7):e6200. doi: 10.1371/journal.pone.0006200 (PMC2703805; doi:10.1371/journal.pone.0006200)

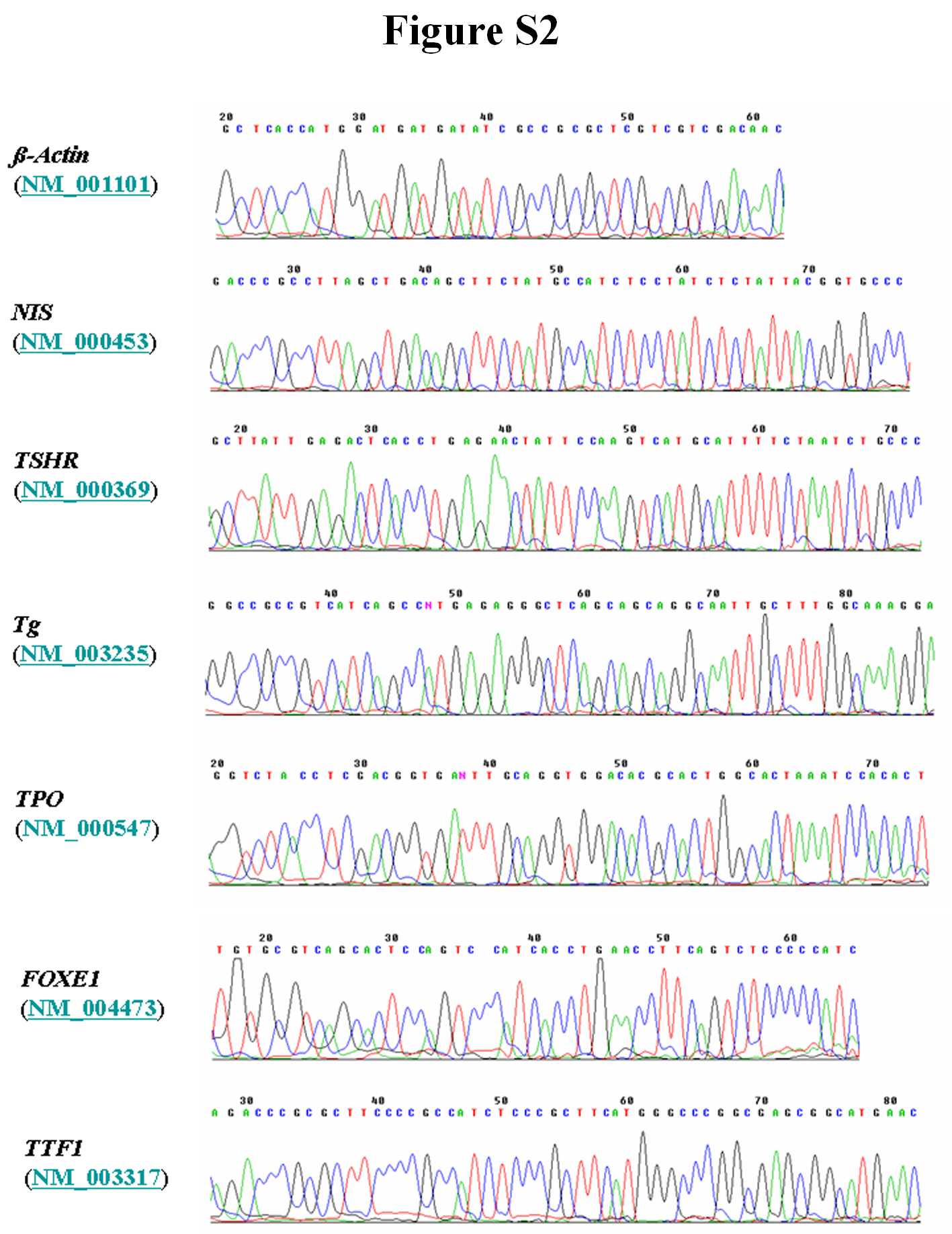

Supplement: Figure S2 — Specificity analysis of real-time quantitative PCR for all investigated genes by sequencing. (1.48 MB TIF) [file pone.0006200.s005.tif]

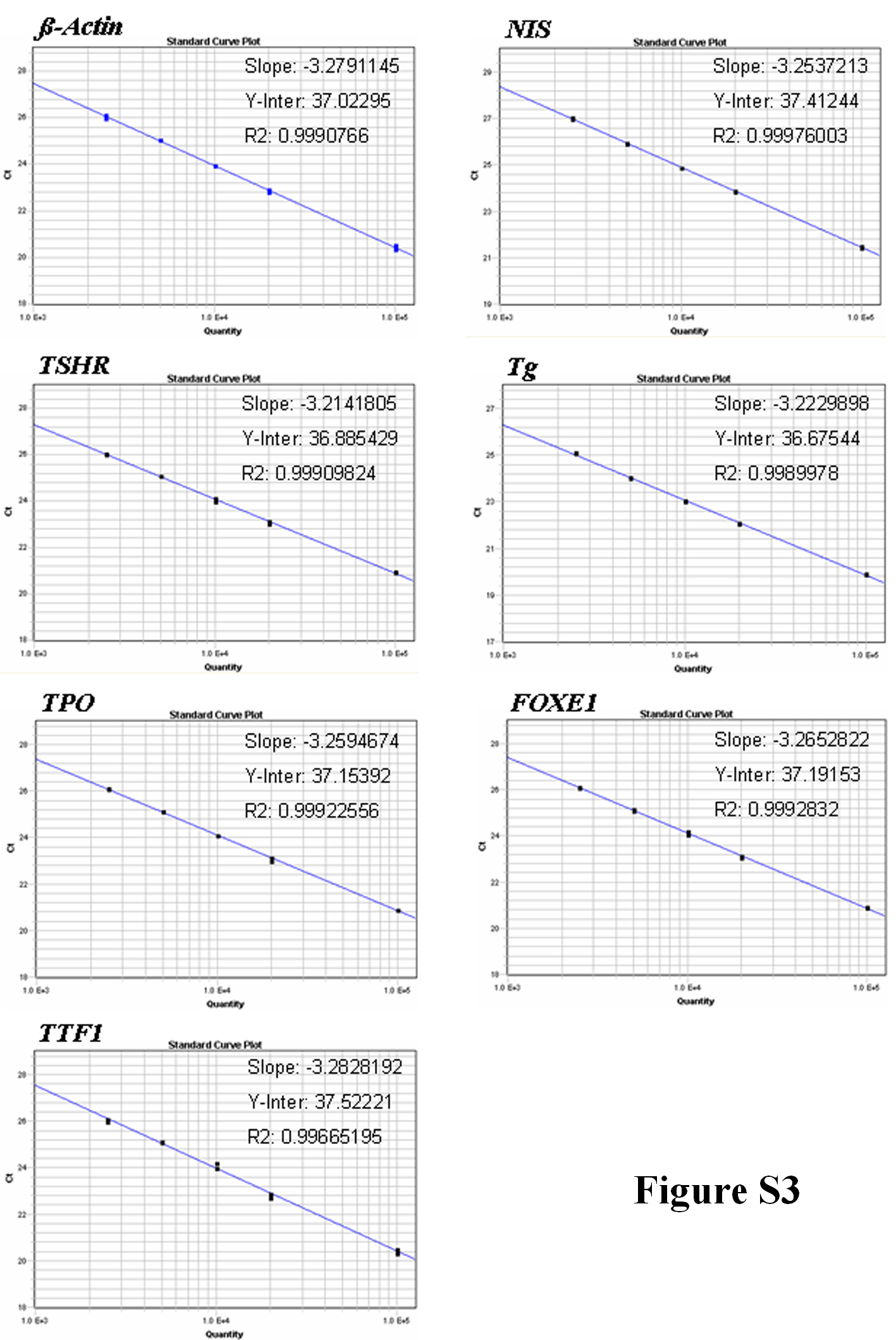

Supplement: Figure S3 — Efficiency evaluation of real-time quantitative PCR for all investigated genes by standard curves. (1.32 MB TIF) [file pone.0006200.s006.tif]
